# Atrial fibrillation and what else?

**DOI:** 10.1007/s12471-022-01668-w

**Published:** 2022-02-25

**Authors:** S. M. van den Bogaard, F. E. Vervaat, P. H. van der Voort

**Affiliations:** Department of Cardiology, Catharina Hospital, Eindhoven, The Netherlands

A 56-year-old male with no pertinent medical history was referred to the emergency department with dyspnoea and fever due to COVID-19 pneumonia. At presentation oxygen saturation was 88%, necessitating supplemental oxygen and hospital admission. Due to a fast and irregular pulse, an electrocardiogram was recorded, which revealed atrial fibrillation (AF) but no other abnormalities (Fig. 1a). The patient experienced no symptoms associated with AF and the duration of AF was unknown. Metoprolol 50 mg twice daily was initiated with the aim of rate control. To achieve better rate control metoprolol was uptitrated to 100 mg thrice daily and digoxin was added. However, after higher doses of metoprolol and digoxin, rhythm monitoring showed repetitive bursts of wide complex tachycardia (Fig. 1b).

**Fig. 1:**
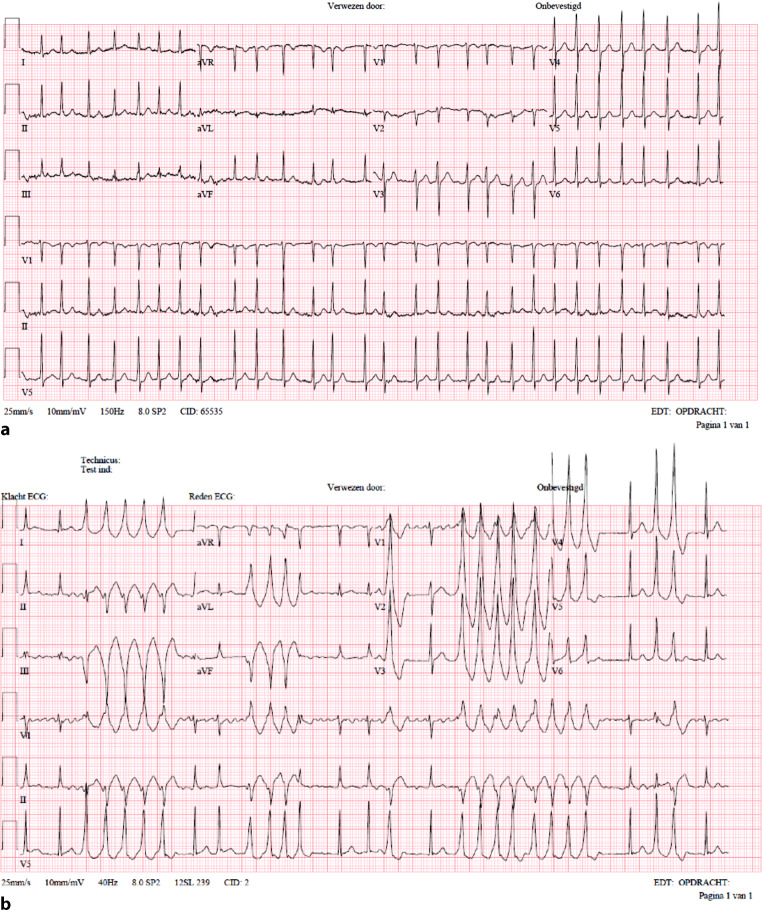
**a** ECG at hospital admission showing atrial fibrillation; **b** ECG with repetitive bursts of wide complex tachycardia after metoprolol and digoxin. *ECG* electrocardiogram

What is your diagnosis based on these electrocardiograms?

## Answer

You will find the answer elsewhere in this issue.

